# The Lithuanian version of the Burnout Assessment Tool (BAT-LT): psychometric characteristics of the primary and secondary symptoms scales

**DOI:** 10.3389/fpsyg.2023.1287368

**Published:** 2023-11-20

**Authors:** Jurgita Lazauskaitė-Zabielskė, Arūnas Žiedelis, Rita Jakštienė, Ieva Urbanavičiūtė, Hans De Witte

**Affiliations:** ^1^Organizational Psychology Research Center, Vilnius University, Vilnius, Lithuania; ^2^Research Group Work, Organizational and Personnel Psychology WOPP−O2L, Faculty of Psychology and Educational Sciences-KU Leuven, Leuven, Belgium; ^3^Optentia Research Unit, North-West University, Vanderbijlpark, South Africa

**Keywords:** burnout assessment tool, BAT, psychometric properties, Lithuania, burnout

## Abstract

The Burnout Assessment Tool (BAT) is a new measure of burnout that was developed to address the shortcomings of existing burnout instruments. This study investigates the psychometric properties of the Lithuanian version of the Burnout Assessment Tool (BAT-LT). In total, 408 adult workers were surveyed (the mean age was 35.94 years; 68.6 per cent were female; 43.9 per cent held managerial positions). Participants came from different sectors of economic activity. The results showed that BAT-LT had good factorial validity, indicating that BAT-LT’s four subscales (exhaustion, cognitive impairment, emotional impairment, and mental distance) can be combined into a single burnout score. Moreover, Cronbach’s alpha values indicate good reliability for all six core and secondary burnout symptoms scales. Furthermore, the results confirmed that BAT-LT could be differentiated from job boredom, workaholism, work engagement and depression. Finally, measurement invariance across managerial status and the sector was observed. The results of this study provide solid evidence for BAT-LT’s reliability and factorial and construct validity.

## 1 Introduction

Burnout refers to a work-related strain resulting from prolonged exposure to chronic stressors of the job (Maslach et al., 2001). It is related to a wide range of adverse outcomes, including lower job satisfaction, organizational commitment (Alarcon, 2011), higher performance, and absenteeism (Swider and Zimmerman, 2010). Moreover, burnout is regarded as contagious and can be passed on among employees (Bakker et al., 2007), supervisors and employees (Huang et al., 2016), and intimate partners (Maslach, 2003; Bakker, 2009). Given the adverse effects of burnout, the WHO (2019) has added it to the International Classification of Diseases, defining it as an occupational phenomenon that influences health. Employers must regularly assess psychosocial risks among their employees and take measures to prevent burnout (Eurofound, 2018). Therefore, valid measures of burnout are needed for researchers and as well as practitioners to assess it correctly.

The Maslach Burnout Inventory (MBI; Maslach and Jackson, 1981) is considered the most popular burnout measure among the many developed burnout assessment instruments. However, as argued by Schaufeli and De Witte (2023), MBI has certain conceptual, technical, and practical limitations. From a conceptual point of view, MBI covers a professional efficacy component, albeit research indicates that this is either an antecedent or a consequence of burnout but not its core dimension (Schaufeli and Taris, 2005). Moreover, MBI does not incorporate reduced cognitive performance despite research supporting the inclusion of it as a component of burnout (Deligkaris et al., 2014). Some other issues, such as item wording and very skewed answers, may also challenge the reliability of the MBI (Bresó et al., 2007; Wheeler et al., 2011; Schaufeli et al., 2020b). Finally, the applicability of the MBI in practice is limited because it does not allow for calculating a total burnout score (Schaufeli et al., 2020b). Considering these limitations, Schaufeli et al. (2020a,b) have developed an alternative measure of burnout−the Burnout Assessment Tool (BAT).

Notably, BAT also differs from other popular measures of burnout. For example, Kristensen et al. (2005) argued that three dimensions are too many to characterize burnout, thereby reducing burnout to one dimension, tapping physical and mental fatigue and exhaustion. They developed the Copenhagen Burnout Inventory (CBI) that does not allow distinguishing between the different aspects of burnout. Moreover, CBI measures personal, work-related and client-related burnout, extending burnout to the non-work domain. This idea has been severely criticized by Schaufeli and Taris (2005) due to collapsing the new term of burnout with the old and well-known concept of fatigue.

Furthermore, another popular instrument for measuring burnout is the Oldenburg Burnout Inventory (OLBI, Demerouti et al., 2003), which assesses the two dimensions of burnout, i.e., exhaustion and disengagement. This instrument uses not only negative but also positive items to assess burnout. As argued by González-Romá et al. (2006), the positive framing of burnout taps into its opposite−work engagement−and, therefore, is likely problematic.

Considering that BAT is a promising instrument in the field of occupational health, the current study adds to the international efforts aimed at validating BAT and investigates some of the psychometric properties of the Lithuanian version of this tool.

### 1.1 The burnout assessment tool

The BAT defines burnout as a syndrome consisting of four interrelated core symptoms that refer to a single underlying psychological construct (Schaufeli et al., 2020a,b). Following the conceptualization of burnout developed by Schaufeli and Taris (2005), who relied on the ideas of the grand old man of psychological fatigue research, Thorndike (1914), suggesting that the basic tenet of fatigue is “the intolerance of any effort,” Schaufeli et al. (2020a,b) have proposed exhaustion and mental distancing that reflect the inability and unwillingness to expend the efforts at work. Exhaustion denotes “a severe loss of energy that results in feelings of both physical and mental exhaustion” (p. 4, Schaufeli and De Witte, 2023). Mental distance pertains to “strong reluctance or aversion, indifference, and cynicism” (p. 4, Schaufeli and De Witte, 2023). Moreover, they have also included cognitive impairment and emotional impairment. Emotional impairment refers to “intense emotional reactions such as anger or sadness, and feeling overwhelmed by one’s emotions,” while cognitive impairment consists of “memory problems, attention and concentration deficits, and poor cognitive performance” (p. 4, Schaufeli and De Witte, 2023).

Additionally, the BAT contains a secondary-symptoms scale with two factors: psychological complaints (e.g., sleep problems, tension, and worrying) and psychosomatic complaints (e.g., headaches, chest and muscle pain). The core symptoms can be measured with a long-form (23 items) or a short-form (12 items) of the instrument, while the secondary-symptoms scale consists of 10 items.

Since the BAT conceptualizes burnout as a syndrome, it implies that the BAT should produce a composite score that indicates the burnout syndrome and different scores that indicate each of the four symptoms. By testing a four-factor model that assumes the BAT to consist of four correlating subscales against a second-order model that assumes all four subscales load on a common higher burnout factor, the existing research shows the latter (which agrees with the notion of a burnout syndrome) to fit better than the four-dimensional model. This was confirmed in employee samples from Austria, Finland, Belgium, Germany, Ireland, Japan, The Netherlands (De Beer et al., 2020), Brazil, Portugal (Sinval et al., 2022), Italy (Consiglio et al., 2021), Poland (Basińska et al., 2021), and Ecuador (Vinueza-Solórzano et al., 2021). Moreover, several studies (De Beer et al., 2020; Sinval et al., 2022) showed that second-order factor structure of the BAT is invariant across countries. These findings suggest that the BAT assesses burnout similarly in particular countries, and it can be used for reliable and valid cross-national comparisons.

Furthermore, the internal consistency of the BAT was tested in various countries such as Austria, The Netherlands, Ireland, Belgium (Flanders), Finland, Germany (De Beer et al., 2020), Japan (Sakakibara et al., 2020), Italy (Consiglio et al., 2021), Ecuador (Vinueza-Solórzano et al., 2021), Korea (Cho, 2020), Brazil and Portugal (Sinval et al., 2022), and Romania (Oprea et al., 2021). These studies show BAT and its subscales to have good reliability, with Cronbach α coefficients exceeding 0.80 for the subscales and 0.90 for the total scale.

Finally, existing research demonstrates the discriminant validity of BAT. For example, based on the Average Variance Extracted, Schaufeli et al. (2020a) demonstrated the BAT to be distinct from job boredom (Dutch Boredom Scale, DUBS; Reijseger et al., 2013), depressed mood (Four-Dimensional Symptom Questionnaire, 4DSQ; Terluin et al., 2006), workaholism (Dutch Workaholism Scale, DUWAS; Rantanen et al., 2015), and work engagement (Utrecht Work Engagement Scale, UWES; Schaufeli et al., 2006). The same results were obtained for the short version of BAT (BAT-12) in a Romanian sample (Oprea et al., 2021). Yokoyama et al. (2022) found workaholism to be positively and work engagement negatively related to burnout measured with the BAT, directly and indirectly through self-endangering behavior. These results indicate that burnout, as assessed with the BAT, can indeed be differentiated from workaholism and work engagement. Pereira et al. (2021) showed that burnout, measured with the BAT, can be distinguished from work-related quality of life and mental health symptoms and mediates the relationship between these two concepts.

### 1.2 The present study

With the current study, we add to the scientific effort of establishing a valid measurement of burnout by evaluating the psychometric properties of the Lithuanian version of the BAT. Although burnout is not recognized as an occupational disease in Lithuania, the existing research shows that various professional groups, especially in the healthcare sector, face a high risk of burnout in Lithuania. For example, in the study by Vaičienė et al. (2022), 70.1 per cent of midwives reported work-related burnout, while Šiupšinskienė et al. (2022) found 82.5 per cent of otorhinolaryngologists were at high risk of burnout. Therefore, measuring burnout and promoting public health policies in the workplace are essential issues for the Lithuanian workforce. Because there is no established instrument or procedure to assess burnout in Lithuania, it is crucial to validate a tool that can be used for screening burnout in occupational settings. The validation of the Lithuanian version of BAT (BAT-LT) is the first step in this direction.

Therefore, in the present study, we investigated the construct validity of the Lithuanian translation of the long version BAT. The first aim was to test whether the data collected with the Lithuanian translation of the questionnaire would reveal the four interrelated theoretically based dimensions of exhaustion, mental distance, emotional impairment, and cognitive impairment. Moreover, we aimed to test the factor structure of secondary burnout symptoms. Furthermore, we explored the discriminant validity of the BAT by testing the relationships between burnout, work engagement, depressed mood, and job boredom.

With this study, we also aimed to contribute to international efforts to validate the BAT. In addition to verifying the theoretical structure of the scale, it is crucial to determine whether the structure holds the same across different contexts and cultures. As burnout is an essential topic for many employees, it is also important to take a closer look at the BAT scores’ invariance concerning employees’ background characteristics. If not, we cannot be sure we are measuring the same construct and even what construct we are measuring. So far, only a few studies examined factorial group invariance. For example, the BAT was found to be invariant across countries (De Beer et al., 2020; Sinval et al., 2022), gender and age (Hadz̆ibajramović et al., 2022).

Moreover, De Beer et al. (2022) showed the BAT to have strong measurement invariance for gender and ethnicity in a South African sample. However, to the best of our knowledge, no study has tested the invariance across job status and sector, which represent important occupational characteristics. Therefore, we tested the invariance of the BAT-LT across managerial status (managers vs. non-managers) and sector (public vs. private). We believe this analysis is essential as testing measurement invariance is a prerequisite to evaluating group differences (for instance, mean differences across managerial status/sector) (Vandenberg and Lance, 2000). In this respect, the research suggests that managers face a high risk of burnout (Blom et al., 2016) as they experience broader and higher job demands (Sirén et al., 2018). Likewise, private sector employees report higher burnout levels (Tsigilis et al., 2006). Hence, we believe the investigation of measurement invariance of the BAT managerial status and sector to be an important step in the validation process.

## 2 Materials and methods

### 2.1 Participants and procedure

A convenience sample of the Lithuanian working population (*N* = 408) was surveyed via an online survey website. To recruit the participants, we relied on student research assistants (Demerouti and Rispens, 2014). Prospective participants were contacted through email and invited to participate in the study. The participants filled out an informed consent form before they completed the questionnaires. Moreover, the participants were informed that the data would be treated anonymously and used only for scientific purposes. Participation in the study was voluntary and gratuitous, with the right to withdraw at any moment.

A total of 68.6 (*n* = 280) per cent of the sample were female, and 30.4 (*n* = 124) were male, while 1 per cent (*n* = 4) did not indicate their gender. The mean age of the sample was 35.94 (SD = 12.56) years, ranging from 16 to 64, and their mean tenure was 6.78 (SD = 8.66) years, ranging from 1 month to 45 years. The majority of the sample (71.8 per cent) had higher education, 6.9 per cent had professional education, 21.1 per cent had secondary education, and 0.2 had primary education. A total of 43.9 per cent (*n* = 179) were managers. On average, the participants worked 38.23 h/week (SD = 16.74).

A total of 60.8 per cent (*n* = 248) of respondents had a full-time job, the rest (39.2 per cent) indicated that their job is part-time. A total of 53.9 per cent (*n* = 220) of the sample worked in a private, and the rest (46.1 per cent) in the public sector. The sample characteristics by industry sector are presented in Table 1.

**TABLE 1:** Overview of the Lithuanian sample.

	Frequency	Per cent
**Industry sector**		
Education and science	96	23.5
Finance and insurance	22	5.4
Information and communication, IT	31	7.6
Public administration and defense	5	1.2
Health care and social work	35	8.6
Wholesale and retail trade	38	9.3
Manufacturing	18	4.4
Electricity, gas, water supply, waste management	5	1.2
Construction, transport and storage, logistics	49	12.0
Artistic, entertainment and recreational activities	33	8.1
Activities of accommodation and catering services	27	6.6
Other	49	12.0

### 2.2 Measures

Respondents were asked to provide demographic data (age, gender, education, managerial status and job type, industry sector) and fill out a questionnaire including items to assess burnout, boredom, engagement, and depressed mood.

**Burnout** was measured with the Burnout Assessment Tool (BAT), developed by Schaufeli et al. (2020b). A total of 23 items (BAT-C) are used to measure four core symptoms of burnout: exhaustion (8 items, e.g., “After a day at work, I find it hard to recover my energy”), mental distance (5 items; e.g., “I feel a strong aversion toward my job”), cognitive impairment (5 items; e.g., “At work I struggle to think clearly”), and emotional impairment (5 items; e.g., “I do not recognize myself in the way I react emotionally at work”). Ten items (BAT-S) are used to measure secondary symptoms: psychological complaints (5 items, e.g., “I tend to worry”) and psychosomatic complaints (5 items, e.g., “I suffer from headaches”). All items were rated on a five-point frequency Likert scale ranging from 1 - never to 5 - always. Responses were summed and averaged for each subscale. BAT was translated from English to Lithuanian by applying a back-translation procedure to ensure that all items were consistent with their original meaning (Brislin, 1980). The items were then further approved by the Lithuanian language editor.

**Boredom** was measured using three items from the Dutch Boredom Scale (Reijseger et al., 2013). Sample item: “During work time, I daydream.” The items were rated on a 7-point Likert scale ranging from 0−never to 6−always/daily.

**Depressed mood** was measured with six items developed by Terluin et al. (2006). Sample item: “During last week, have you felt that everything is meaningless?” The items were rated on a five-point Likert-type scale, ranging from 1 - not at all to 5 - very often or constantly. Responses were summed and averaged for the scale.

**Work engagement** was assessed with a 3-item ultra-short Utrecht work engagement scale, developed by Schaufeli et al. (2017) and validated in Lithuania (Lazauskaitė-Zabielskė et al., 2020). The scale includes three items measuring vigor, dedication, and absorption. The items were rated on a 7-point Likert scale ranging from 0−never to 6−always/daily. Responses were summed and averaged for the scale, as Schaufeli et al. (2006) recommended.

### 2.3 Data analyses

SPSS-28 software was used for descriptive data and reliability analyses. Confirmatory factor analyses (CFA), construct validity and measurement invariances analyses were conducted using Amos 29.0.

We carried out confirmatory factor analyses for several different models using maximum likelihood estimation. We analyzed core and secondary burnout symptoms separately. To assess the goodness of fit, we used different indicators. A good fit exists when the comparative fit index (CFI) and the Tucker-Lewis index (TLI) are at least higher than 0.90, preferably higher than 0.95 (Hu and Bentler, 1995, p. 76–99); while the root mean square error of approximation (RMSEA) is 0.08 or less (Byrne, 2011, p. 73). Akaike information criterion (AIC) has no absolute cut-off scores, but it was used to compare alternative models, with lower values showing a better fit (Burnham and Anderson, 2004).

Four models that include the core symptoms of the BAT were tested: (1) a 1-factor model in which all 23 items load on one latent factor; (2) a 4-factor correlated model, in which the four main aspects (mental distance, exhaustion, cognitive impairment and emotional impairment) are separate but intercorrelated; (3a) a second-order model where four core aspects load on one latent factor; this model is based on the approach that burnout is one underlying psychological state or disorder consisting of four types of complaints; (3b) a second-order model as in (3a) step, but with adjustments that emerged during the analyses – adding a correlation between the residuals of items 16 and 18 of the Cognitive Impairment aspect of the burnout. If a second-order model is confirmed, then both a single score of the BAT and four separate scores for distinct aspects can be used for assessments.

Two models were tested concerning the secondary burnout symptoms: (1) a 1-factor model, in which all ten items load on one single factor; (2) a correlated 2-factor model in which psychological distress and psychosomatic complaints each load on a separate factor. A second-order model with only two factors is not identified mathematically and, therefore, not tested.

To test scale reliability, we evaluated the internal consistency of the BAT core symptoms scale, all of its subscales and the internal consistency of two secondary symptoms using Cronbach’s α. Evaluations of alpha exceeding 0.7 show an acceptable fit, > 0.8−a good fit, and > 0.9−an excellent fit (George and Mallery, 2003, p. 231). Additionally, composite reliability was evaluated for the BAT core symptoms scale. Composite reliability uses factor loadings instead of item covariances (Padilla and Divers, 2016), allowing better estimations for non-congeneric items with different factor loadings. Values between 0.7 and 0.9 are evaluated as ranging from satisfactory to good (Hair et al., 2021, p. 77). An online tool (Colwell, 2016), which used the mathematical formula by Raykov (1997), was used to obtain the value of composite reliability.

Moreover, we assessed construct validity in terms of convergent and discriminant validity, following the guidelines of Fornell and Larcker (1981). Convergent validity reflects the level to which the construct converges in order to explain the variance of its indicators. For this evaluation, average variance extracted (AVE) is used, and it is equivalent to the communality of a construct. The minimal acceptable AVE is 0.5, which would specify that a construct explains 50% of the indicators’ variance (Hair et al., 2021, p. 77).

Furthermore, the discriminant validity of the BAT was assessed using comparisons to other aspects of work-related well-being, i.e., work engagement, boredom at work, and depressed mood. As per the guidelines of Fornell and Larcker (1981), the construct’s AVE (burnout’s AVE) should be juxtaposed to the squared inter-construct correlation of that same construct and all other constructs in the structural model (work engagement, boredom at work, and depressed mood in our analysis). The squared inter-construct correlation is a measure of shared variance between two constructs, and it should not be larger than their AVEs for discriminant validity to be present.

Finally, the BAT measure was inspected for multigroup measurement invariance. More specifically, we tested configural, metric, scalar, and residual invariance (Putnick and Bornstein, 2016) to establish the equivalence of model form, factor loadings, intercepts, and residual errors in different groups of employees according to managerial status (managers vs. non-managers) and sector (public vs. private). Metric invariance was observed if the Comparative Fit Index (CFI) decreased by less than 0.01, the Root Mean Square Error of Approximation (RMSEA) increased by less than 0.015, and the chi-square increased non-significantly after imposing constraints on factor loadings (Chen, 2007). Similarly, scalar invariance was confirmed if changes in fit indices were not larger than indicated after imposing constraints on intercepts, and residual invariance was established if such changes did not occur after constraining residual errors to be equal among groups.

## 3 Results

### 3.1 Factorial validity

Table 2 shows the goodness-of-fit indices of the analyzed models for the core symptoms of burnout. As expected, the 1-factor model indices did not show a good fit. An adjusted 4-factor model and an adjusted second-order model fit well with the data, indicating that the four aspects of burnout can be distinguished. When comparing TLI, CFI, RMSEA and its confidence intervals, χ^2^ and degrees of freedom changes related to the aforementioned two adjusted models, none of these measures clearly indicate which model should be preferred. AIC supports the adjusted 4-factor model as somewhat better, yet the overall fitting of both models seems to be good, and the second-order adjusted model cannot be rejected. This means that both a single score of the BAT and four separate scores for distinct aspects can be used for evaluations. However, as Schaufeli et al. (2020a) argue, it is essential to highlight that, with the confirmed second-order model, burnout can be interpreted as a syndrome comprising related symptoms that appear due to one underlying psychological condition. In the four-factor model, the correlations among factors were moderate to strong, ranging from 0.57 to 0.76.

**TABLE 2:** Fit indices of the CFA of the core dimensions of the BAT-LT (*N* = 408).

Model		*χ* ^2^	df	Δ*χ*^2^	*p* for Δ*χ*^2^	CFI	TLI	AIC	RMSEA (90%CI)
1	1-factor model	1713.70	230	-		0.71	0.68	1805.70	0.13 (0.12; 0.13)
2a	4-factor model	696.61	224	1017.09 (from 1)	<0.001	0.91	0.90	846.61	0.07 (0.07; 0.08)
2b	Adjusted 4-factor model	659.48	223	37.13 (from 2a)	<0.001	0.92	0.90	811.48	0.07 (0.06; 0.08)
3a	Second-order model	700.66	226	4.05 (from 2a)	>0.05	0.91	0.90	846.66	0.07 (0.07; 0.08)
3b	Adjusted second-order model	662.93	225	33.68 (from 2a) 3.45 (from 2b)	<0.001; >0.05	0.92	0.90	819.93	0.07 (0.06; 0.08)

*χ*^2^, chi-square; df, degrees of freedom; CFI, Comparative fit index; TLI, Tucker-Lewis index; AIC, Akaike information criterion; RMSEA, Root mean square error of approximation, CI, confidence intervals. All χ^2^ values are significant at *p* < 0.001. Adjusted models include a correlation between the residuals of items 16 and 18 (of the Cognitive Impairment aspect of the burnout).

In the adjusted second-order model, the item loadings on the four factors ranged from 0.48 (item No. 2) to 0.86 (item No. 17), with item No. 2 being the only one with a loading below 0.5. The average loading was 0.72, also showing good convergent validity. All factor loadings of the core dimensions and their items can be seen in Table 3. Three dimensions of general burnout, namely, exhaustion, mental distance and cognitive impairment had a very good factor loading, ranging from 0.83 to 0.9, and emotional impairment had a slightly lower loading of 0.67. The original and the Lithuanian version of the BAT is presented in the Supplementary material.

**TABLE 3:** Factor loadings of the core dimensions and their items.

Item	Standardized factor loading	Subscale	Standardized factor loading (on General burnout)
1	0.69	Exhaustion	0.83
2	0.48		
3	0.78		
4	0.67		
5	0.70		
6	0.68		
7	0.63		
8	0.76		
9	0.74	Mental distance	0.90
10	0.56		
11	0.78		
12	0.79		
13	0.66		
14	0.84	Cognitive impairment	0.84
15	0.84		
16	0.79		
17	0.86		
18	0.65		
19	0.78	Emotional impairment	0.67
20	0.79		
21	0.61		
22	0.79		
23	0.76		

The 2-factor model fits the data better than the 1-factor model, confirming the two distinct aspects of the secondary symptoms (see Table 4). All fit indices and AIC support the 2-factor model, with a significant (*p* < 0.001) Δχ^2^ between the 1- and 2-factor models. The correlation between the two factors in the CFA was *r* = 0.72, showing a strong relationship.

**TABLE 4:** Confirmatory factor analysis of the secondary symptoms of the BAT-LT.

Model		*χ* ^2^	df	Δ*χ*^2^	p for Δ*χ*^2^	CFI	TLI	AIC	RMSEA (90%CI)
1	1-factor model	254.84	35	−	−	0.87	0.83	294.84	0.12 (0.11; 0.14)
2	2-factor model	95.99	34	1	<0.001	0.96	0.95	137.99	0.07 (0.05; 0.08)

N, 408. *χ*^2^, chi-square; df, degrees of freedom; CFI, Comparative fit index; TLI, Tucker-Lewis index; AIC, Akaike information criterion; RMSEA, Root mean square error of approximation. CI, confidence intervals. Both χ^2^ values are significant at *p* < 0.001.

The item loadings on the 2-factors were in the proper range from 0.55 (item No. 24) to 0.85 (item No. 25). All factor loadings of the secondary dimensions’ items can be seen in Table 5.

**TABLE 5:** Factor loadings of the secondary dimensions’ items.

Item	Standardized factor loading	Subscale
24	0.55	Psychological complaints
25	0.85	
25	0.85	
27	0.80	
28	0.58	
29	0.69	Psychosomatic complaints
30	0.75	
31	0.61	
32	0.65	
33	0.58	

### 3.2 Reliability

The four core symptoms of burnout were closely related, correlation coefficients ranging from 0.50 to 0.65. Moreover, these four aspects were also associated with the secondary symptoms, yet the coefficients were slightly smaller, ranging from 0.40 to 0.57 (see Table 6).

**TABLE 6:** Correlations between the burnout symptoms and reliability indices.

	Scale	CR	Cronbach’s α	1	2	3	4	5	6
1	BAT core symptoms	0.89	0.93	–					
2	Exhaustion	0.87	0.87	0.87	–				
3	Mental Distance	0.84	0.83	0.84	0.65	–			
4	Emotional Impairment	0.86	0.86	0.75	0.51	0.50	–		
5	Cognitive Impairment	0.90	0.90	0.82	0.59	0.65	0.53	–	
6	Psychological Distress	0.85	0.84	0.62	0.57	0.43	0.53	0.49	–
7	Psychosomatic complaints	0.79	0.80	0.57	0.51	0.40	0.49	0.45	0.62

All correlations are significant at *p* < 0.001.

All core and secondary symptoms of burnout had Cronbach’s alphas in the range of 0.8–0.9, indicating a very good fit and internal consistency. The BAT scale’s Cronbach’s α score for the core symptoms was 0.93, also indicating a reliable measure.

Composite reliability scores for the core symptoms were between 0.84 and 0.90, and secondary symptoms indices were 0.79 and 0.85, all showing good internal consistency (Hair et al., 2021, p. 77).

### 3.3 Construct validity

As Table 7 shows, convergent validity was observed, as the average variance extracted from burnout was 0.66, well above the minimal threshold of 0.5 (Hair et al., 2021, p. 77). The BAT explained 66% of the indicators’ variance. The discriminant validity of the BAT was evaluated by comparing its average variance extracted to squared latent correlations with other work-related constructs of work engagement, boredom at work, and depressed mood. Burnout’s AVE was juxtaposed to the squared inter-construct correlations (R^2^), and they were all smaller than the burnout’s AVE (see Table 7). This confirms that the BAT measures a different concept−that is, burnout−than instruments that measure other work-related well-being constructs.

**TABLE 7:** Average variance extracted (AVE), squared latent correlations (R^2^), and Cronbach’s α for work engagement, boredom, depressed mood, and burnout.

Scale	AVE		R^2^		
		**Cronbach’s α**	**Work engagement**	**Burnout**	**Boredom**
Work engagement	0.70	0.86	–	–	–
Burnout	0.66	0.93	0.40	–	–
Boredom	0.55	0.75	0.17	0.49	–
Depressed mood	0.68	0.93	0.22	0.35	0.22

In Table 7 it can also be seen that other variables in this study−boredom, depressed mood, and work engagement−have appropriate Cronbach’s αs (0.75–0.93).

Discriminant validity was also confirmed by evaluating correlations among constructs of interest. Shapiro-Wilk test indicated that data representing burnout, work engagement, boredom and depressed mood are not normally distributed, hence Spearman correlation coefficients were used to evaluate the relations among the aforementioned constructs. Results are shown in Table 8. As expected, burnout had a moderate negative relation to work engagement and moderate positive to depressed mood and boredom, thus confirming discriminant validity.

**TABLE 8:** Correlations of burnout, work engagement, depressed mood and boredom scales.

Scale	Burnout	Work engagement	Depressed mood
Work engagement	−0.49	–	–
Depressed mood	0.52	−0.39	–
Boredom	0.46	−0.32	0.35

All correlations are significant at *p* < 0.001.

### 3.4 Measurement invariance

First, we tested for measurement invariance among employees who held managerial versus non-managerial positions. Results are presented in Table 9. As can be seen, the fit of the data to the model did not significantly deteriorate after the factor loadings were constrained to be equal for managers and non-managers. Changes were also below the indicated threshold when the intercepts were constrained to be equal. Finally, after constraints were imposed so that the residual errors did not differ between groups, the data fit the theoretical structure similarly well. Thus, residual invariance was fully confirmed.

**TABLE 9:** Measurement invariance by managerial status.

	χ^2^ (df)	CFI	RMSEA (90%CI)
Configural invariance model	988.46(450)	0.90	0.05 (0.05; 0.06)
Metric invariance model	1010.68(472)	0.90	0.05 (0.05; 0.06)
Scalar invariance model	1044.89(495)	0.90	0.05 (0.05; 0.06)
Residual invariance model	1064.48(518)	0.90	0.05 (0.05; 0.06)

df, degrees of freedom; CI, confidence intervals. All χ^2^ values are significant at *p* < 0.001.

Similar results were obtained when assessing invariance between workers in different sectors (see Table 10). Residual invariance was also confirmed, which means that public and private sector employees have a similar understanding of the questionnaire items.

**TABLE 10:** Measurement invariance by sector.

	χ^2^ (df)	CFI	RMSEA (90%CI)
Configural invariance model	981.12(450)	0.90	0.05 (0.05; 0.06)
Metric invariance model	996.97(472)	0.90	0.05 (0.05; 0.06)
Scalar invariance model	1029.76(495)	0.90	0.05 (0.05; 0.06)
Residual invariance model	1058.81(518)	0.90	0.05 (0.05; 0.06)

df, degrees of freedom; CI, confidence intervals. All χ^2^ values are significant at *p* < 0.001.

## 4 Discussion

The present study contributes to the international validation of a new burnout assessment measure, the BAT (Schaufeli et al., 2020b), by testing its psychometric properties in a heterogeneous convenience sample of Lithuanian employees. The risk of burnout is prevalent in the world of work, surrounded by a variety of stressors, and it is considered a major challenge for employees’ health and well-being (Demerouti et al., 2021). While severe forms of the burnout syndrome are relatively uncommon, international data suggests that a larger share of the workforce may experience it to a milder degree (Eurofound, 2018). Therefore, there is a need for valid and up-to-date instruments that allow for the assessment of key symptoms indicating this detrimental psychological state in different linguistic and occupational contexts.

In the current study, we report the results from the first validation of the BAT in the Lithuanian language setting. According to our findings, the BAT-LT has good to excellent psychometric properties in terms of structure, construct validity, and reliability of the scale scores. Our analyses support both a correlated four-factor model and a second-order model consisting of four lower-order factors that represent the core symptoms of burnout (i.e., exhaustion, cognitive and emotional impairment, and mental distancing). This is in line with the conceptualization of burnout as a syndrome within the framework of the BAT (Schaufeli et al., 2020b) and extends prior international efforts to demonstrate such structure across cultures (e.g., De Beer et al., 2020; Sinval et al., 2022). Notably, not all prior studies have tested both of the above factor model specifications. Drawing on the conceptualization of burnout by Schaufeli et al. (2020b), some studies have focused on investigating a higher-order model. For instance, De Beer et al. (2020) found that a higher-order core symptom factor structure holds well across seven countries. Studies that have tested different model specifications, have found similar goodness of fit indices for both a correlated four-factor model and a higher-order model (e.g., Sakakibara et al., 2020; Schaufeli et al., 2020a). Therefore, the current findings are in line with those observed in previous validation research conducted in other countries. Our results showed that all items effectively represented their respective factors, as indicated by high factor loadings. In the present study, the lowest standardized factor loading was observed in the exhaustion subscale (λ_EX2_ = 0.48), which is similar to what has been documented in some previous research (e.g., De Beer et al., 2022). Each of the burnout core symptom subscales also demonstrated good internal consistency, as evidenced by high scale reliability coefficients. Furthermore, we were able to empirically support a two-factor structure for the secondary symptoms of burnout, consisting of psychological and psychosomatic complaints. As expected, the secondary symptoms were positively correlated with the core symptoms of burnout. While the correlations were moderate in strength (i.e., ranging from.40 to.57), we did not observe a major overlap with the core symptoms, which aligns with the original theorizing behind the BAT.

Such findings have both research and practical implications. Our study adds to the cross-cultural burnout literature by showing that the construct is perceived in a similar way (i.e., it retains the same structure) yet in another linguistic context that is quite different from English. Translation of measures into other languages is considered a specific challenge in psychological research and assessment practice, as linguistically bound differences in item interpretation may contribute to measurement non-invariance (Flake et al., 2022). The present findings indicate that, in a configural sense, the BAT-LT operates similarly to its original version, enabling a clear identification of the core dimensions of burnout. From a research perspective, this implies enhanced cross-cultural comparability and replicability of findings, which is important considering the global research efforts devoted to studying this phenomenon.

Moreover, our findings lend support to the discriminant validity of the construct. This means that, as measured with the BAT-LT, burnout can be distinguished from other psychological states such as depressed mood, job boredom, and work engagement. Psychological constructs often have blurry boundaries, and this issue hinders the interpretation of measurements. For example, burnout symptoms have been shown to overlap with depressive symptoms, especially when using earlier conceptualizations of the construct (e.g., Bianchi et al., 2015). The current validation results are encouraging, suggesting that the BAT, based on an updated four-dimensional view of burnout, may provide the much-needed precision in differentiating its symptoms from those of other stress-related states. This is important not only from a practical perspective but also in developing further research on the topic. As seen in recent studies on work-related well-being, there is increased attention to understanding the co-occurrence of burnout (or its specific dimensions) with job boredom and work engagement (e.g., Moeller et al., 2018; Harju et al., 2023) as well as their inter-relationships with each other (e.g., Junker et al., 2021; Sousa and Neves, 2021). Therefore, high quality measurement tools are of utmost importance for answering these questions.

Last but not least, in the present study we tested how the BAT-LT works in different occupational settings, examining the measurement invariance with regard to employees’ managerial status and organizational sector. Our findings support strict invariance, which indicates that the burnout items were perceived in a similar way irrespective of our participants’ hierarchical position and sector they were working in. This is an important psychometric aspect considering that most research samples are heterogeneous and include participants with diverse characteristics. Establishing measurement invariance can thus provide with greater confidence and precision when comparing burnout’s measurement scores across the above-mentioned occupational groups. While in recent years the BAT has been cross-validated in a number of different languages (e.g., De Beer et al., 2020), additional equivalence testing has mostly focused on employees’ demographic characteristics (De Beer et al., 2022; Hadz̆ibajramović et al., 2022). By focusing on occupational groups our findings provide supplementary evidence about the measurement quality of the instrument. In this way, they are significant not only to the Lithuanian researcher and occupational health practitioner community, but also contribute to the international body of research dedicated to developing a valid burnout screening tool.

## 5 Limitations and future research directions

While the Lithuanian version of the BAT seems to have good psychometric properties, the present study is not exempt from certain limitations. First, it was based on a convenience sample, which is not necessarily representative of the entire working population in Lithuania. Further studies might consider retesting the BAT-LT on a larger scale, which would also allow for inspecting its psychometrics across a wider range of employee subpopulations and socio-demographic groups.

Moreover, the current study uses a cross-sectional design. Therefore, we were not able to test certain aspects that require longitudinal data, such as longitudinal invariance. Since these aspects are important for both research and practice, future studies should pay special attention to them.

A further analysis of the nomological network of the BAT-LT is also pertinent in order to elaborate on construct validity. It would allow researchers to obtain a more fine-grained view of where burnout (as measured with the BAT) stands in relation to other related constructs, such as psychosocial job characteristics or psychological states, as well as to what extent it is related to indicators of burnout measured with other instruments.

Finally, a cautionary note must be made to occupational health professionals interested in using the BAT for assessment or diagnostic purposes. Given the promising results on the validity and reliability of the instrument, establishing national norms and cut-off scores is an important next step in the development of this instrument, as noted by Schaufeli et al. (2023). Research in this direction is currently in progress.

## 6 Conclusion

The present study provides support for the reliability and validity of burnout scores, as assessed using the Lithuanian version of the BAT. According to the findings, the BAT-LT had favorable psychometric properties in terms of factor structure, construct validity, and internal consistency of the scales. Notably, our study has verified the four dimensions of burnout and supported the burnout syndrome idea based on a higher-order factor structure. This leads to the possibility to use both a total score and sub-scores, when needed.

Moreover, the BAT-LT offers possibility to differentiate between core and secondary symptoms of burnout (based on correlational data) while also displaying discriminant validity by distinguishing burnout from other psychological states. In addition, we observed strict measurement invariance across participants’ occupational characteristics, such as managerial status and sector. As a result, we maintain that the BAT-LT represents a promising new tool for measuring the burnout syndrome in Lithuania.

## Data availability statement

The raw data supporting the conclusions of this article will be made available by the authors, without undue reservation.

## Ethics statement

Ethical review and approval was not required for the study on human participants in accordance with the local legislation and institutional requirements. Written informed consent from the patients/participants or patients/participants legal guardian/next of kin was not required to participate in this study in accordance with the national legislation and the institutional requirements.

## Author contributions

JL-Z: Conceptualization, Methodology, Writing−original draft, Writing−review and editing. AŽ: Data curation, Formal analysis, Methodology, Writing−review and editing. RJ: Data curation, Formal analysis, Methodology, Writing−review and editing. IU: Conceptualization, Methodology, Writing−original draft, Writing−review and editing. HD: Writing−review and editing.
