# Supplementary material for: The Lithuanian version of the Burnout Assessment Tool (BAT-LT): psychometric characteristics of the primary and secondary symptoms scales
Source: Front Psychol. 2023 Nov 20;14:1287368. doi: 10.3389/fpsyg.2023.1287368 (PMC10698373; doi:10.3389/fpsyg.2023.1287368)
Supplement: Supplementary file 1 [file Table_1.docx]

Supplementary Material

**The Lithuanian version of the burnout assessment tool (BAT-LT): psychometric characteristics of the primary and secondary symptoms scales**

**Jurgita Lazauskaitė-Zabielskė*, Arūnas Žiedelis, Rita Jakštienė, Ieva Urbanavičiūtė, Hans De Witte**

*** Correspondence:** Jurgita Lazauskaitė-Zabielskė: jurgita.lazauskaite@fsf.vu.lt

**The original and the Lithuanian version of the BAT**

***Core Symptoms***

| No if Item | ***Exhaustion*** | ***Išsekimas*** |
| --- | --- | --- |
| 1 | At work, I feel mentally exhausted* | Darbe jaučiuosi protiškai išsekęs (-usi)* |
| 2 | Everything I do at work requires a great deal of effort | Viskas, ką darau darbe, reikalauja didelių pastangų |
| 3 | After a day at work, I find it hard to recover my energy* | Po darbo dienos man sunku atgauti jėgas* |
| 4 | At work, I feel physically exhausted* | Darbe jaučiuosi fiziškai išsekęs (-usi)* |
| 5 | When I get up in the morning, I lack the energy to start a new day at work | Atsikėlus ryte man trūksta jėgų pradėti naują darbo dieną |
| 6 | I want to be active at work, but somehow I am unable to manage | Noriu būti aktyvus (-i) darbe, bet man tai kažkaip nepavyksta |
| 7 | When I exert myself at work, I quickly get tired | Kai įtemptai dirbu, greitai pavargstu |
| 8 | At the end of my working day, I feel mentally exhausted and drained | Darbo dienos pabaigoje jaučiuosi protiškai išsekęs (-usi) ir išsunktas (-a) |
| ***Mental distance*** | | ***Psichologinis atsiribojimas*** |
| 9 | I struggle to find any enthusiasm for my work* | Sunkiai randu noro dirbti* |
| 10 | At work, I do not think much about what I am doing and I function on autopilot | Dirbdamas (-a) pernelyg negalvoju, ką darau, ir dirbu autopilotu |
| 11 | I feel a strong aversion towards my job* | Nekenčiu savo darbo* |
| 12 | I feel indifferent about my job | Esu abejingas (-a) savo darbui |
| 13 | I’m cynical about what my work means to others* | Netikiu, kad mano darbas svarbus kitiems* |
| ***Cognitive impairment*** | | ***Kognityvinė žala*** |
| 14 | At work, I have trouble staying focused* | Darbe man sunku išlaikyti dėmesį* |
| 15 | At work I struggle to think clearly* | Darbe man sunku aiškiai mąstyti |
| 16 | I’m forgetful and distracted at work | Darbe būnu užmaršus (-i) ir išsiblaškęs (-usi) |
| 17 | When I’m working, I have trouble concentrating* | Dirbant man sunku susikaupti* |
| 18 | I make mistakes in my work because I have my mind on other things | Dirbdamas (-a) darau klaidas, nes galvoju apie kitus dalykus* |
| ***Emotional impairment*** | | ***Emocinė žala*** |
| 19 | At work, I feel unable to control my emotions* | Jaučiu, kad darbe man sunkiai sekasi kontroliuoti savo emocijas* |
| 20 | I do not recognize myself in the way I react emotionally at work* | Darbe taip emociškai sureaguoju, kad neatpažįstu savęs* |
| 21 | During my work I become irritable when things don’t go my way | Darbe susierzinu, kai kas nors vyksta ne taip, kaip norėčiau |
| 22 | I get upset or sad at work without knowing why | Darbe susinervinu ir nusimenu be priežasties |
| 23 | At work I may overreact unintentionally* | Darbe netyčia galiu perdėtai emociškai reaguoti* |

*Note:* * = Short version

***Secondary Symptoms***

| No if Item  ***Psychological complaints*** | | ***Psichologiniai nusiskundimai*** | |  |
| --- | --- | --- | --- | --- |
| 1 | I have trouble falling or staying asleep | Man sunku užmigti arba išmiegoti visą naktį | |  |
| 2 | I tend to worry | Aš linkęs (-usi) nerimauti | |  |
| 3 | I feel tense and stressed | Jaučiu įtampą ir stresą | |  |
| 4 | I feel anxious and/or suffer from panic attacks | Jaučiu nerimą arba patiriu panikos priepuolių | |  |
| 5 | Noise and crowds disturb me | Mane trikdo triukšmas ir minia | |  |
| ***Psychosomatic complaints*** | | | ***Psichosomatiniai nusiskundimai*** | |
| 6 | I suffer from palpitations or chest pain | Mane vargina smarkus širdies plakimas ar skausmai krūtinėje | |  |
| 7 | I suffer from stomach and/or intestinal complaints | Mane vargina skrandžio ir/arba žarnyno negalavimai | |  |
| 8 | I suffer from headaches | Mane vargina galvos skausmai | |  |
| 9 | I suffer from muscle pain, for example, in the neck, shoulder or back | Mane vargina raumenų skausmai, pavyzdžiui, kaklo, pečių ar nugaros | |  |
| 10 | I often get sick | Aš dažnai sergu | |  |
